# Asperolide A prevents bone metastatic breast cancer via the PI3K/AKT/mTOR/c‐Fos/NFATc1 signaling pathway

**DOI:** 10.1002/cam4.3432

**Published:** 2020-09-25

**Authors:** Wenli Jiang, Youlutuziayi Rixiati, Hao Huang, YiJun Shi, Caiguo Huang, Binghua Jiao

**Affiliations:** ^1^ Department of Biochemistry and Molecular Biology College of Basic Medical Navy Medical University Shanghai China; ^2^ Department of Pathology Soochow University Medical School Suzhou China; ^3^ Department of Orthopaedics Shaoxing People's Hospital Shaoxing China

**Keywords:** Asperolide A, bone metastases, breast cancer, mTOR, osteoclast

## Abstract

**Background:**

Breast cancer is the leading cause of death among women with malignant tumors worldwide. Bone metastasis is the main factor affecting the prognosis of breast cancer. Therefore, both antitumor and anti‐breast‐cancer‐induced osteolysis agents are urgently needed.

**Methods:**

We examined the effect of Asperolide A (AA), a marine‐derived agent, on osteolysis and RANKL‐induced phosphoinositide 3‐kinase (PI3K)/AKT/mTOR/c‐FOS/nuclear factor‐activated T cell 1 (NFATc1) pathway activation, F‐actin ring formation, and reactive oxygen species (ROS) generation in vitro. We evaluated AA effect on breast cancer MDA‐MB‐231 and MDA‐MB‐436 cells in vitro through CCK8 assay, wound healing assay, transwell assay, Annexin V‐FITC/PI staining for cell apoptosis, and cell cycle assay. Furthermore, we assessed the effect of AA in vivo using a breast cancer‐induced bone osteolysis nude mouse model, followed by micro‐computed tomography, tartrate‐resistant acid phosphatase staining, and hematoxylin and eosin staining.

**Results:**

Asperolide A inhibited osteoclast formation and differentiation, bone resorption, F‐actin belt formation, ROS activity, and osteoclast‐specific gene and protein expressions and prevented PI3K/AKT/mTOR/c‐FOS/NFATc1 signaling activation in a dose‐dependent manner in vitro. AA also inhibited breast cancer growth and breast cancer‐induced bone osteolysis by reducing osteoclast formation and function and inactivated PI3K/AKT/mTOR signaling in vivo.

**Conclusions:**

Our study demonstrated that AA suppressed bone metastatic breast cancer. These findings indicate AA as a potential, novel curative drug candidate for patients with bone metastatic breast cancer.

## Graphical abstract

Our study showed that Asperolide A (AA) repressed breast cancer‐induced osteoporosis. These findings indicate AA as a potential, novel curative drug candidate for breast cancer patients with bone metastases.

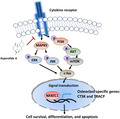

## 1 INTRODUCTION

Breast cancer is the main cause of cancer‐related death among women according to global cancer statistics in 2018.^1^ Although the diagnosis and the treatment of breast cancer have been greatly improved, there is still no significant augmentation in patient overall survival due to the high likelihood of distant metastasis.^2^, ^3^, ^4^ Clinical data showed that bone is the most common site of metastasis in breast cancer and occurs in 65%‐75% of breast cancer,^5^, ^6^ leading to bone pain and pathological bone fractures.^7^

Previous studies have identified an interplay between breast cancer cells and monocyte/macrophage precursor cells.^8^ Metastatic breast cancer cells secrete receptor activator of nuclear factor kappa‐B (NF‐κB) ligand (RANKL) to osteoclast formation and function. Matured osteoclasts in turn release cytokines to promote the development of breast cancer.^8^, ^9^ The phosphoinositide 3‐kinase (PI3K)‐AKT‐mTOR signaling pathway is associated with the development of cancer and osteolysis in vivo and in vitro.^10^ Inhibition of the PI3K‐AKT‐mTOR signaling pathway can suppress breast cancer growth and breast cancer‐induced osteoclast formation. Increased levels of reactive oxygen species (ROS) can also promote osteoclast formation and activation,^11^ and ROS levels are higher in tumor cells than in normal cells.^12^ ROS is a key marker in tumor therapy. Thus, novel therapeutic agents that inhibit the PI3K‐AKT‐mTOR/c‐Fos/nuclear factor‐activated T cell 1 (NFATc1) signal pathways and ROS level are urgently needed.

Tetranorditerpenoids wentilactones have shown good antitumor activities. We previously extracted and isolated Asperolide A (AA), Wentilactone A (WA), and Wentilactone B (WB) from the marine algae‐derived endophytic fungus *Aspergillus wentii* EN‐48.^13^, ^14^, ^15^, ^16^, ^17^ We assayed the cytotoxic activities against NCI‐H460, Hela, SMMC‐7721, and SW1990 cancer cell lines.^13^ In current study, we demonstrated that AA‐induced cell cycle arrest and cell apoptosis, inhibited metastasis, and prevented invasiveness. More importantly, low‐dose AA was found to inhibit RANKL‐induced osteoclast activity in vitro and breast cancer‐induced osteolysis in vivo. Additionally, we demonstrated the effects of AA on PI3K/AKT/mTOR/c‐Fos/NFATc1 signaling pathways.

## 2 MATERIALS AND METHODS

### 2.1 Materials and reagents

Asperolide A, WA, and WB were extracted and isolated from the marine algae‐derived endophytic fungus *A wentii* EN‐48. BEZ235 was purchased from Selleck. All the compounds were dissolved in 100% dimethyl sulfoxide and stored at −20°C until use. Mouse macrophage‐colony stimulating factor (M‐CSF) and RANKL were purchased from R&D Systems. The Cell Cycle and Apoptosis Analysis Kit, penicillin/streptomycin, Triton X‐100, 4% paraformaldehyde (PFA), phenylmethylsulfonyl fluoride (PMSF), protease inhibitors, bicinchoninic acid (BCA) protein assay kit, Tris‐buffered saline‐tween‐20 (TBST), and phosphatase inhibitors were acquired from Beyotime. The complete alpha modification (α‐MEM) culture medium, fetal bovine serum (FBS), phosphate‐buffered saline (PBS), Dulbecco's modified Eagle medium (DMEM), and Leibovitz's L‐15 medium (L‐15) were obtained from Gibco (Thermo Fisher Scientific). The cell counting kit‐8 (CCK8) was purchased from Dojindo Molecular Technology. The tartrate‐resistant acid phosphatase (TRAcP) dyeing kit and NH_4_OH were purchased from Sigma‐Aldrich. The Carboxy‐H2DFFDA kit was purchased from Solarbio Science & Technology Co., Ltd. Phalloidin‐iFluor 488 reagent and 4′6‐diamidino‐2‐phenylindole (DAPI) were purchased from Abcam. Alkaline phosphatase (ALP) and the Alizarin Red staining (ARS) kit were from Nanjing Jiancheng Chemical Industrial Co. Ltd. The RNeasy Mini kit was from Qiagen. The PrimeScript RT Master Mix Kit was from Takara Bio. Nitrocellulose (NC) membranes were from Millipore. The Trans‐Blot^®^ TurboTM system was from Bio‐Rad. Specific primary antibodies against β‐actin, mTOR, P‐mTOR(S2481), PI3K, P‐PI3K(Thr308), AKT, P‐AKT(Ser473), c‐Fos, NFATc1, ALP, RUNX2, and osteocalcin were acquired from Abcam. P‐P65, P65, P‐JNK, JNK, P‐ERK, ERK, P38, and P‐P38 were purchased from Cell Signaling Technology.

### 2.2 Cell culture

Bone marrow macrophages (BMMs) were derived from the long bones of 4‐week‐old C57BL/6 mice (the Chinese Academy of Sciences) according to the previous methods^18^, ^19^ and seeded in special culture medium (50 ng/mL M‐CSF, 1% penicillin/streptomycin, 10% FBS in complete α‐MEM). The culture medium was replaced every 2 days. Adherent BMMs at this stage, which depended on M‐CSF, were osteoclast precursors.

MDA‐MB‐231 cells were cultured in DMEM and MDA‐MB‐436 cells were cultured in Leibovitz's L‐15 with 1% penicillin/streptomycin and 10% FBS under 5% CO_2_ at 37°C. The culture medium was replaced every 2 days.

### 2.3 CCK8 assay

Cells were seeded into 96‐well plates at 4 × 10^4^ cells/mL, cultured for 24 hours, and then treated with WA, WB, or AA for 48 or 96 hours. Cells were then incubated with 10 μL CCK8 reagent without light for 1.5 hours at 37°C. The absorbance at 450 nm was measured on a BioTek Elx808 absorbance microplate reader (BioTek Instruments). Each condition was repeated in triplicate and cell viability was calculated using GraphPad Prism version 7.

### 2.4 TRAcP staining

Bone marrow macrophages were seeded into a 96‐well plate at a density of 8 × 10^3^ cells/well in special culture medium (50 ng/mL M‐CSF, 50 ng/ml RANKL, 1% penicillin/streptomycin, 10% FBS) in complete α‐MEM and AA, WA, or WB. The culture medium containing AA, WA, or WB was replaced every 2 days until multinucleated osteoclasts formed. After osteoclasts formed, the cells were then fixed with 4% PFA for 10 minutes and then stained by TRAcP dyeing solution. TRAcP‐positive multinucleate cells with three or more nuclei were scored as osteoclast (OCL)‐like cells. Images were captured using a Nikon Eclipse TS100 inverted routine microscope (Nikon Instruments Inc). The number of TRAcP‐positive cells was counted and the area of the osteoclast was calculated using ImageJ software version 1.8.0 (NHI).

### 2.5 Bone resorption pit formation assay

Bone marrow macrophages were plated on 96‐well bone‐mimicking hydroxyapatite‐coated OsteoAssay culture plates (Corning Inc) at a density of 8 × 10^3^ cells/well (in triplicate). The cells were incubated with M‐CSF (50 ng/mL) and RANKL (50 ng/mL), and then adding various concentrations of AA (0, 0.5, or 1 μmol/L) after mature osteoclast formation. The culture medium was replaced every 2 days for 8‐10 days in a 5% CO_2_ cell incubator at 37°C. Adherent osteoclasts were removed with NH_4_OH (0.25 mol/L) for 1 minute and then washed by distilled water two times. Finally, the areas of the bone resorption pits were calculated using ImageJ software.

### 2.6 Intracellular ROS activity assay

Intracellular ROS production was measured using the Carboxy‐H2DFFDA kit according to the manufacturer's protocol. BMMs were plated in a 96‐well plate at 8 × 10^3^ cells/well in culture medium (50 ng/mL M‐CSF, 50 ng/mL RANKL, 1% penicillin/streptomycin, 10% FBS) along with various concentrations of AA for 48 hours. BMMs were washed by PBS and then incubated with carboxy‐H_2_DCFDA staining solution for 1.5 hours at 37°C. The cells were then washed with PBS in the dark. Fluorescence images were captured using a Cytation 5 Cell Imaging Multi‐Mode Reader (BioTek Instruments), and ROS production was measured using ImageJ software.

### 2.7 F‐actin ring formation assay

Osteoclasts derived from BMMs were induced by RANKL and treated without or with different AA concentrations (0, 0.5, or 1 μmol/L) until mature osteoclast cells were formed in the control wells. Cells were fixed with 4% PFA for 15 minutes, permeated with 0.5% Triton X‐100, washed with PBS, and then incubated with Phalloidin‐iFluor 488 reagent for 50 minutes at 4°C without light. Cells were washed again with PBS and then incubated with DAPI for 5 minutes at room temperature (RT) without light. Fluorescence images were captured by a LSM5 confocal microscope and measured using Zeiss ZEN software. The area of the F‐actin ring was calculated using ImageJ software.

### 2.8 Cell apoptosis assay

Apoptosis was evaluated using the Cell Cycle and Apoptosis Analysis Kit. Cells were digested by trypsin, washed once with PBS, and resuspended in 1× Annexin‐binding buffer. Early apoptosis was detected via Alexa Fluor^®^488 Annexin V and propidium iodide (PI) staining. Stained cells were evaluated using a CytoFLEX flow cytometer (Beckman Coulter Inc) and data were acquired using Cell Quest software, version 3.0.

### 2.9 Cell cycle assay

Cell cycle assay was performed using the Cell Cycle and Apoptosis Analysis Kit. Cells were harvested, washed once with PBS, and fixed in 75% precooling ethanol for 24 hours at 4°C. Cells were then washed with PBS two times and incubated with RNase A and PI reagents for 30 minutes at 37°C. Cell cycle analysis was performed using a CytoFLEX flow cytometer. Data were acquired using Cell Quest software, version 3.0.

### 2.10 Transwell invasion assay

Transwell invasion assay was performed as described in a previous study.^20^ Cells (in triplicate) were seeded in the 24‐well upper chambers with 8 μmol/L pore; the bottom chamber contained 500 μL conditioned medium. Cells were washed once with PBS and then stained with crystal violet dye. Images were captured using a Nikon Eclipse TS100 inverted routine microscope (Nikon Instruments Inc). The number of the invaded cells was calculated using Image J software.

### 2.11 Wound healing assay

Cells (10^6^ cells/well) were seeded into 6‐well plates in culture medium with AA or BEZ235. A scratch was made in the middle of the plate using a 200‐μL pipette tip. Images were captured at 0 and 12 hours. The area percentage of wound healing was analyzed using ImageJ software.

### 2.12 ALP staining and ARS

Bone marrow‐derived stem cells were plated in 24‐well plates in osteogenic differentiation culture medium (10 mmol/L β‐glycerophosphate, 50 μg/mL vitamin C, 1 × 10^−8^ mol/L dexamethasone, 15% FBS, 1% penicillin/streptomycin in DMEM) and stimulated with AA (0.25, 0.5, or 1 μmol/L). After 7 days of staining by ALP and 20 days of staining by ARS, cells were washed two times with PBS and fixed with 4% PFA for 15 minutes. The positive stained area was analyzed using ImageJ software.

### 2.13 Quantitative real time polymerase chain reaction

RNA was extracted from BMM‐derived‐osteoclasts using the RNeasy Mini kit (Qiagen) according to the manufacturer's instructions. cDNA was obtained using a PrimeScript RT Master Mix Kit (Takara Bio, Otsu, Japan). quantitative real time polymerase chain reaction was performed using SYBR Premix Ex Taq^TM^ II, specific forward and reverse primers, and double distilled water on a PRISM 7500 system (ABI). The cycling conditions were as follows: 95°C for 5 minutes, 42 cycles at 95°C for 10 seconds, 60°C for 20 seconds, 72°C for 20 seconds, and the final extension step at 72°C for 20 seconds. The primers were as follows: Cathepsin k (CTSK; Forward: 5′‐GATACTGGACACCCACTGGGA‐3′; Reverse: 5′‐CATTCTCAGACACAATCCAC‐3′), c‐FOS (Forward: 5′‐GCGAGCAACTGAGAAGAC‐3′; Reverse: 5′‐TTGAAACCCGAGAACATC‐3′), TRAP/ACP5 (Forward: 5′‐CTGGAGTGCACGATGCCAGCGACA‐3′; Reverse: 5′‐TCCGTGCTCGGCGATGGACCAGA‐3′), NFATc1 (Forward: 5ʹ‐CCGTTGCTTCCAGAAAATAACA‐3ʹ; Reverse: 5ʹ‐TGTGGGATGTGAACTCGGAA‐3ʹ), and Glyceraldehyde‐3‐phosphate dehydrogenase (GAPDH) (Forward: 5ʹ‐ACCCAGAAGACTGTGGATGG‐3ʹ; Reverse: 5ʹ ‐CACATTGGGGGTAGGAACAC‐3ʹ). The expression of target genes was normalized to the level of GAPDH mRNA. All assays were repeated three times. Gene expression was calculated with 2^−∆∆CT^.

### 2.14 Western blot

Cells were lysed in radioimmunoprecipitation assay lysis buffer supplemented with PMSF, protease and phosphatase inhibitors at 4°C for 30 minutes, followed by centrifugation at 12 000 g for 15 minutes. Protein concentration was determined using the BCA protein assay kit. Protein samples were separated by sodium dodecyl sulfate polyacrylamide gel electrophoresis and then transferred to NC membranes using the Trans‐Blot^®^ Turbo^TM^ system (Bio‐Rad). Nitrocellulose membranes were blocked in TBST supplemented with 5% nonfat milk and incubated with primary antibodies for 8 hours at 4°C. The NC membranes were washed four times with TBST and incubated with fluorescence secondary antibodies for 1.5 hours at RT. Immunogenicity was tested using an Odyssey v3.0 image scanner (LI‐COR Biosciences). Band quantification was analyzed by ImageJ software.

### 2.15 Breast cancer‐induced bone metastasis model

A breast cancer‐induced bone metastasis model was established as described previously.^21^ Healthy female bagg’s albino/c nude mice (n = 24; 8 weeks old) were purchased from the Chinese Academy of Sciences and raised for a week to allow acclimatization to the laboratory environment. Mice were divided into four groups: sham group (n = 6), control group (vehicle, n = 6), low‐dose group (0.5 mg/kg, n = 6), and high‐dose group (2.5 mg/kg, n = 6). MDA‐MB‐231 cells (8 × 10^5^) in 50 μL of PBS were injected into the left tibiae of mice in the control group, low‐dose group, and high‐dose group. At 7 days after the injection, mice received intraperitoneal injection of AA in 15% ethanol, 80% HBSS, and 5% PEG300 daily for 4 weeks. The left legs of mice were collected on day 28 and scanned with a micro‐CT SkyScan1176 scanner (Bruker) at 50 kV/450 μA at a 9‐μmol/L pixel size. Reconstructed images were recombined using CT Vol and CTvox software. Trabecular bone volume fraction (BV/TV%), bone surface/bone volume (BS/BV%), trabecular thickness (Tb.Th), trabecular number (Tb.N), and trabecular separation (Tb.Sp%) were measured using CTAn software. Animal experiments in the study was approved by the Institutional Animal Care and Use Committee of Navy Medical University.

### 2.16 Histological staining, TRAP staining, and Immunohistochemistry (IHC)

The leg samples were decalcified in 10% Ethylene Diamine Tetraacetic Acid for 4 weeks at RT and then processed for embedding in paraffin blocks. Tissues were sectioned into 5‐μm thick slices for TRAcP staining, hematoxylin and eosin (H&E) staining, and IHC. Tibia femurs sections underwent H&E staining. Stained sections were examined using an Eclipse TS100 inverted light microscope (Nikon Instruments Inc). The total number of TRAcP‐positive cells and the osteoclast surface per bone surface (Oc.S/BS) were calculated using ImageJ software. IHC analysis was performed with heat‐induced antigen retrieval in sodium‐citrate buffer (Dako). Primary antibodies used was anti‐phospho‐PI3K at 1:100. Biotinylated secondary antibody used was the EnVision + system HRP kit (Dako), and then nuclei were stained with hematoxylin.

### 2.17 Statistical analyses

All data are presented as mean ± SD (n ≥ 3 per group). Statistical differences were determined using one‐way ANOVA with Dunnett's post hoc test for comparisons between different groups using GraphPad Prism software and SPSS 13.0 software (IBM Corp). *P* < .05 indicated statistical significance between groups.

## 3 RESULTS

### 3.1 AA inhibits RANKL‐induced osteoclastogenesis, suppresses hydroxyapatite resorption, and represses RANKL‐induced ROS production and F‐actin ring formation in vitro

**FIGURE 1:**
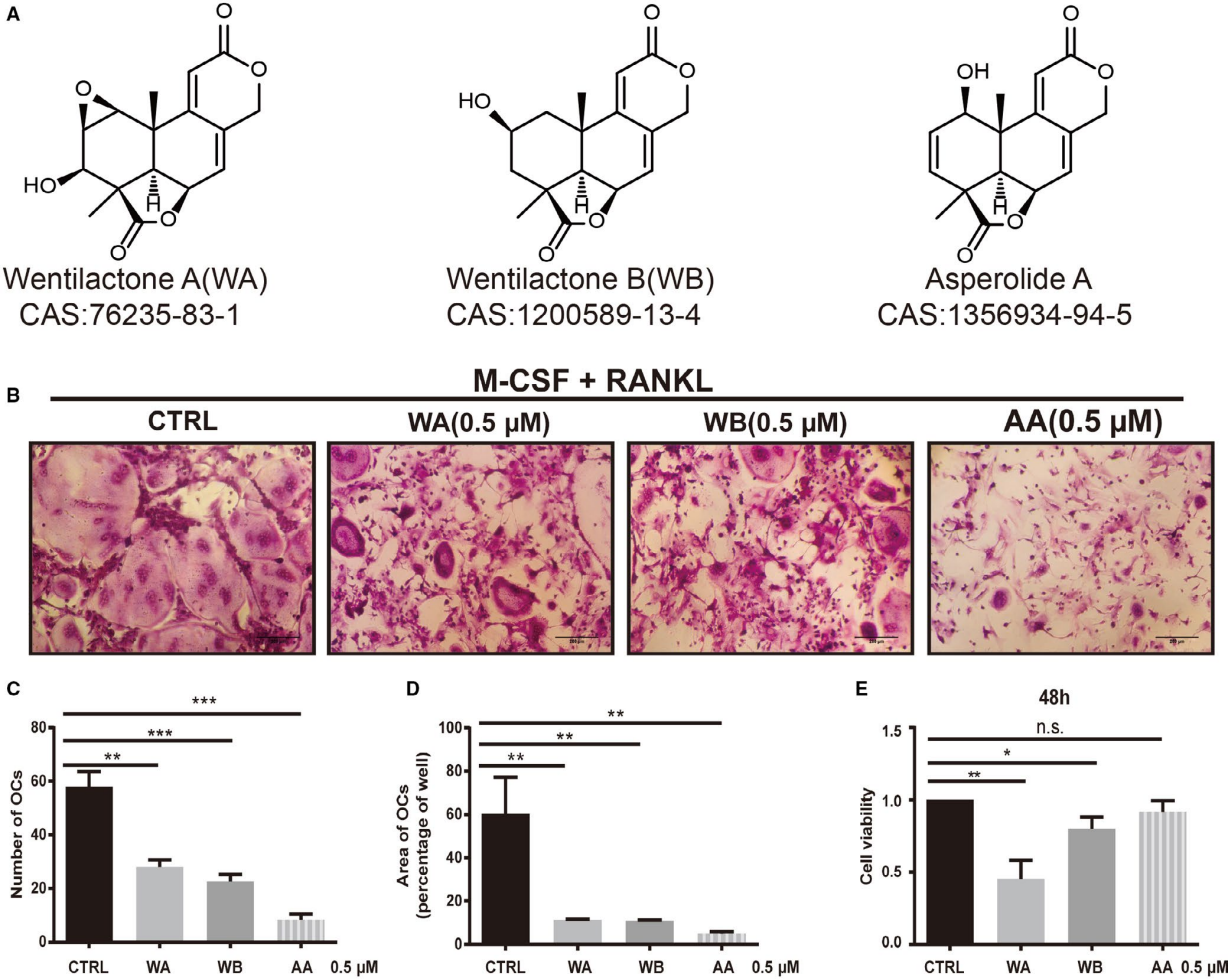
Compounds from the marine algae‐derived endophytic fungus *Aspergillus wentii* EN‐48 inhibited receptor activator of NF‐κB ligand (RANKL)‐induced osteoclast formation with low or no cytotoxicity in vitro. The chemical structure and CAS (Chemical Abstracts Service) number of each compound (A). Macrophage‐colony stimulating factor (M‐CSF)‐dependent bone marrow macrophages (BMMs) were treated with 50 ng/mL RANKL with or without compounds for 5 d. Tartrate‐resistant acid phosphatase (TRAcP) staining was performed until osteoclasts formed (n = 3 per group) (B). The number of TRAcP‐positive osteoclasts (nuclei ≥ 3) was quantified (percentage of control) (C). The area/size of TRAcP‐positive osteoclasts (nuclei ≥ 3) was quantified (percentage of control) (D). The cytotoxic effect of each compound on M‐CSF‐dependent BMMs was measured by CCK8 assay. BMMs were cultured with 50 ng/mL M‐CSF with compounds (0.5 μmol/L) for 48 h (n = 3 per group) (E). Cell viability is expressed as a percentage of control (CTRL). Data are shown as mean ± SD; n = 3 per group; **P < .01 and ***P < .005, compared with untreated groups.

To investigate the effects of marine alga‐derived sesquiterpene lactone compounds on osteoclastogenesis, a cell activity screening was performed. The chemical structures of WA, WB, and AA are shown in Figure 1A. Primary BMMs were treated with RANKL and M‐CSF in the presence of WA, WB, and AA at 0.5 μmol/L for 48 hours. AA showed the most significant inhibition of osteoclast differentiation without toxicity to BMMs when treated at 0.5 μmol/L for 48 hours (Figure 1B‐E)

**FIGURE 2:**
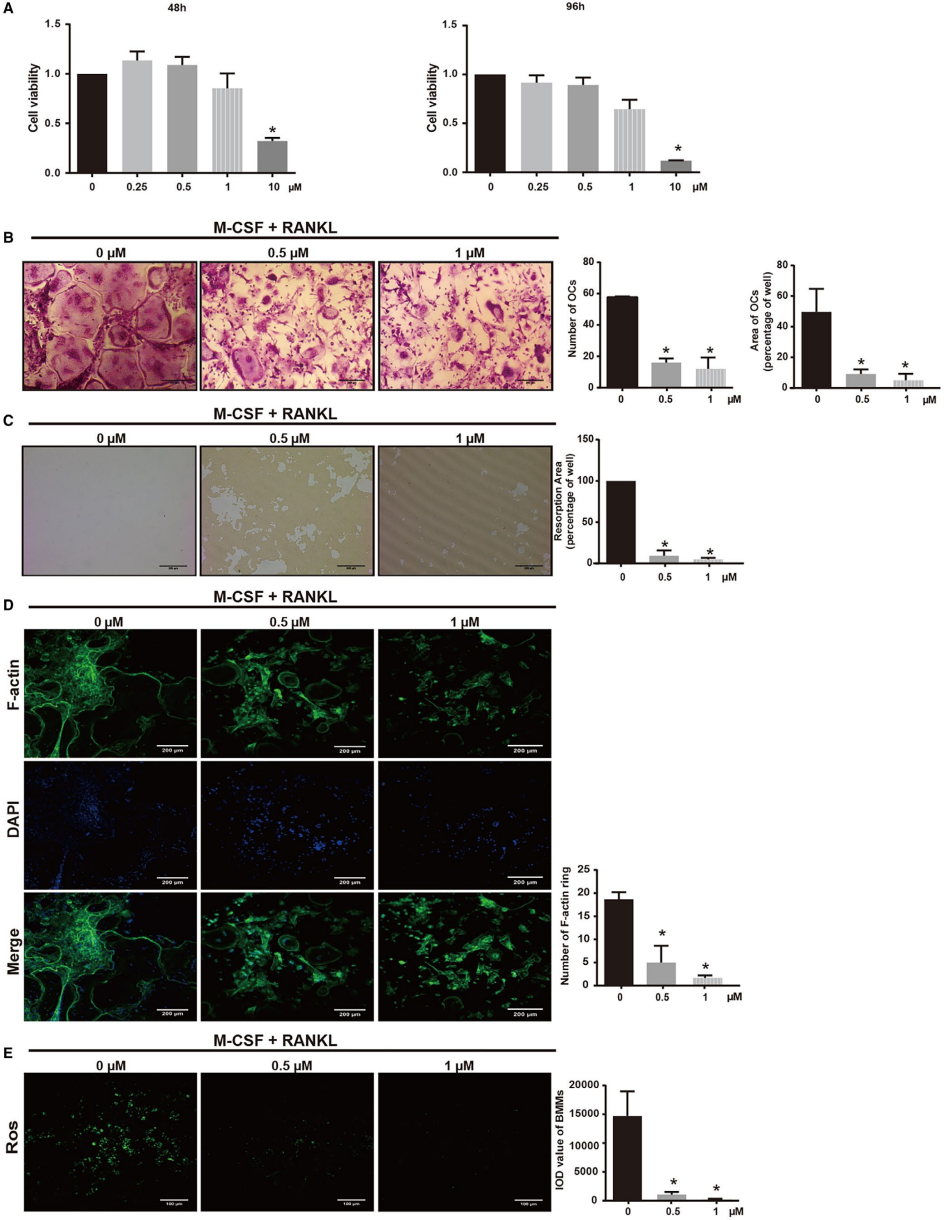
Asperolide A (AA) attenuates receptor activator of NF‐κB ligand (RANKL)‐induced osteoclastogenesis, hydroxyapatite absorption, ROS production, and F‐actin ring formation without cytotoxicity. Effect of AA on macrophage‐colony stimulating factor (M‐CSF)‐dependent bone marrow macrophages (BMM) cell viability by cell counting kit‐8 (CCK8) assay. BMMs were cultured with 50 ng/mL M‐CSF without or with AA (0.25, 0.5, 1, and 10 μmol/L) for 48 and 96 h (n = 3 per group) (A). CCK8 assay was performed. Cell viability is expressed as percentage of the nontreatment group (0 μmol/L). M‐CSF‐dependent BMMs were treated with 50 ng/mL RANKL and then treated without or with AA (0.5 and 1 μmol/L) for 5 days. Cells were fixed and tartrate‐resistant acid phosphatase (TRAcP) staining was performed until osteoclasts formed (n = 3 per group). The number of TRAcP‐positive cells (nuclei > 3) was quantified (n = 3 per group). The area of TRAcP‐positive cells (nuclei > 3) was quantified (n = 3 per group) (B). M‐CSF‐dependent BMMs were treated with AA (0.5 and 1 μmol/L) on bone‐mimicking hydroxyapatite‐coated OsteoAssay culture plates until bone resorption pits were observed. Bone resorption pits were observed using an inverted light microscope. Quantification of resorption area (C). F‐actin ring images from mature osteoclasts were observed using a fluorescence microscope. Quantification of the number of F‐actin rings (D). Intracellular ROS levels of nontreatment and AA‐treated BMMs were detected using the DCFDA fluorescence probe. Integral optic density value reflected the ROS levels in different groups of cells (E). Data are shown as mean ± SD; n = 3 per group; *P < .05, compared with the untreated group cells. ROS, reactive oxygen species. DCFDA, 2ʹ,7ʹ‐dichlorofluorescin diacetate

To confirm the absence of cell toxicity of AA on BMMs, CCK8 assay was performed. The results showed that BMMs viability was not inhibited by AA at concentrations lower than 1 μmol/L at 48 hours (Figure 2A). The IC_50_ for AA in BMMs was 2.22 ± 1.10 μmol/L at 48 hours and 1.89 ± 0.61 μmol/L at 96 hours.

To further investigate the effect of AA on osteoclastogenesis, BMMs were treated with RANKL and M‐CSF in the presence of various concentrations of AA (0, 0.5, 1 μmol/L) during osteoclast formation. As shown in Figure 2B, there were numerous TRAP‐positive multinucleated osteoclasts in the nontreatment group (57.67 ± 0.47 osteoclasts/well), while osteoclast differentiation was suppressed and the number of osteoclasts decreased by up to 79.19% in AA treatment groups (12.00 ± 5.89 osteoclasts/well in the 1 μmol/L AA group, 16.00 ± 2.16 osteoclasts/well in the 0.5 μmol/L AA group.

To identify the effect of AA on osteoclast function, a bone absorption assay was performed. While large bone resorbed areas were observed in the nontreatment group, smaller and fewer bone resorbed areas were observed in the AA treatment groups (Figure 2C). The absorption area decreased to 4.93 ± 1.50% after treatment with 1 μmol/L AA compared with the control group (*P* < .05). We next examined the effect of AA on F‐actin ring formation. As shown in Figure 2D F‐actin ring number and morphology were significantly suppressed by AA at concentrations of 0.5 μmol/L (*P* = .0038) and 1 μmol/L (*P* < .05).

To investigate the effect of AA on RANKL‐induced intracellular ROS production during osteoclastogenesis, ROS levels were examined in RANKL‐stimulated BMMs with the cell permeant oxidation‐sensitive dye carboxy‐H2DCFDA and positive signals were visualized using fluorescence microscopy. The integrated optical density value of BMMs was significantly decreased in the presence of AA in a dose‐dependent manner compared with the nontreatment group (Figure 2E).

Together, these results demonstrated that AA inhibited RANKL‐induced osteoclast formation, intracellular ROS production, and F‐actin ring formation without cytotoxic effects.

### 3.2 AA suppresses osteoclastic‐specific genes and inhibits RANKL‐induced osteoclast formation via the PI3K/AKT/mTOR/c‐Fos/NFATc1 signaling pathway

**FIGURE 3:**
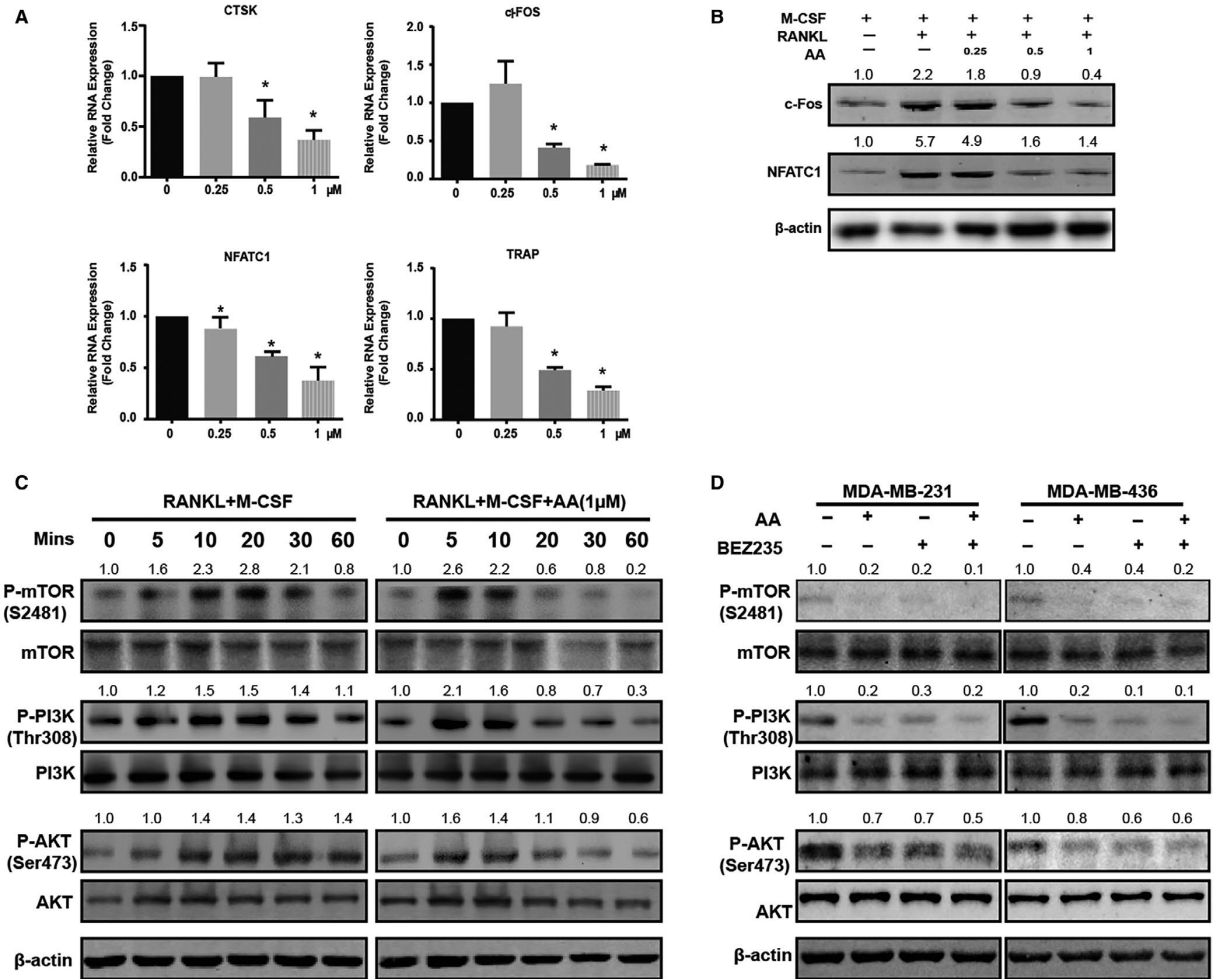
Asperolide A (AA) suppresses osteoclastic‐specific genes and inhibits receptor activator of NF‐κB ligand (RANKL)‐induced osteoclast formation via the PI3K/AKT/mTOR/c‐Fos/NFATc1 signaling pathway. The expressions of cathepsin k (CTSK), c‐FOS, NFATc1, and TRAP genes were examined by quantitative real time polymerase chain reaction (A). The expressions of c‐FOS and NFATc1 proteins were examined by western blot (B). Macrophage‐colony stimulating factor (M‐CSF)‐dependent bone marrow macrophages were plated in 6‐well plates in starvation culture medium for 2 h, pretreated without or with 1 μmol/L AA for 60 min, and then treated without or with 50 ng/mL RANKL for 5, 10, 20, 30, and 60 min. Western blot was performed to evaluate phosphorylated mTOR (P‐mTOR), total mTOR, phosphorylated PI3K (P‐PI3K), total PI3K, phosphorylated AKT (P‐AKT), total AKT, and β‐actin (C). MDA‐MB‐231 and MDA‐MB‐436 cells were plated in 6‐well plates in starvation culture medium for 2 h and pretreated without or with AA and BEZ235 for 60 min. Western blot was performed to detect phosphorylated mTOR (P‐mTOR), total mTOR, phosphorylated PI3K (P‐PI3K), total PI3K, phosphorylated AKT (P‐AKT), total AKT (D). Data are shown as mean ± SD; n = 3 per group; *P < .05

To investigate the effects of AA on osteoclast formation, we evaluated RANKL‐induced osteoclastic‐specific gene expressions in BMMs, such as CTSK, c‐Fos, TRAP, and NFATc1 genes. Quantitative real time polymerase chain reaction results showed that the expressions of all osteoclast‐specific genes examined were inhibited by AA in a dose‐dependent manner (Figure 3A), confirming that AA suppressed osteoclast formation. We also evaluated the levels of osteoclastic‐specific proteins, including c‐Fos and NFATc1, by western blot. Both c‐Fos and NFATc1 protein levels were decreased in BMMs treated with AA at certain concentrations compared with the nontreatment group (Figure 3B).

To further explore the potential mechanism by which AA exerts its inhibitory effect on osteoclast formation, we investigated the effect of AA on the PI3K/AKT/mTOR pathway. Phosphorylation of PI3K, AKT, and mTOR was upregulated by RANKL stimulation of BMMs. However, the activation of PI3K, AKT, and mTOR was significantly inhibited in BMMs by AA treatment (Figure 3C). The PI3K/AKT/mTOR pathway is also a key signaling pathway for tumor development. We found that the activation phosphorylation of PI3K, AKT, and mTOR was significantly decreased after AA treatment in MDA‐MB‐231 and MDA‐MB‐436 breast cancer cells (Figure 3D).

### 3.3 AA inhibits proliferation, migration, and invasion and induces apoptosis and cell cycle arrest of breast cancer cells in vitro

**FIGURE 4:**
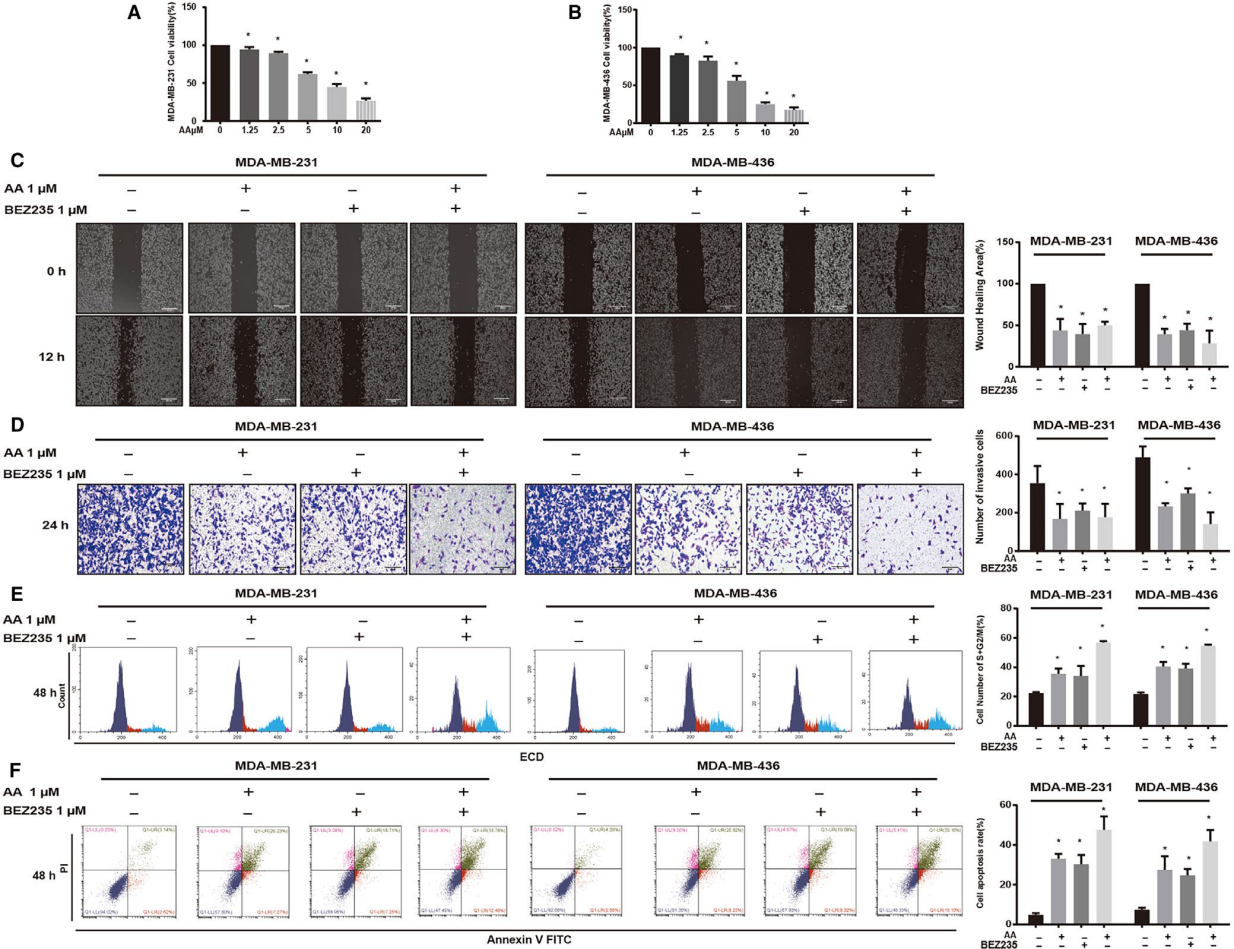
Asperolide A (AA) inhibited the proliferation, migration, and invasion and induced cell cycle arrest and apoptosis of MDA‐MB‐231 and MDA‐MB‐436 cells. Cell viability (%) of nontreatment or AA treatment groups was calculated (A, B). AA inhibited cell migration as determined by wound healing assays (C). Crystal violet‐stained cells were captured and the number of invasive cells was counted (D). AA induced cell cycle arrest in S + G2/M phase and quantification of cells in S + G2/M (%) are shown (E). Apoptosis was determined using Annexin V‐FITC + PI staining and apoptosis rate (%) in each group (F). Data are shown as mean ± SD; n = 3 per group; *P < .05 compared with the untreated group cells

To investigate the effect of AA on MDA‐MB‐231 and MDA‐MB‐436 triple‐negative breast cancer cells, CCK8 assay was performed. Cell viability decreased in response to AA in a dose‐dependent manner; the calculated IC_50_ was 7.61 ± 1.61 μmol/L in MDA‐MB‐231 and 5.04 ± 0.37 μmol/L in MDA‐MB‐436 (48 hours; Figure 4A,B). We next explored the effect of AA on the motility of breast cancer cells. Wound healing assay showed that MDA‐MB‐231 and MDA‐MB‐436 cells migration were decreased after AA treatment. The wound healing area (%) at 12 hours was decreased to 43.99 ± 11.19% in MDA‐MB‐231 and decreased to 39.37 ± 5.00% in MDA‐MB‐436 after treatment with 1 μmol/L AA (Figure 4C). We further found that AA blocks breast cells invasion. The number of invading MDA‐MB‐231 and MDA‐MB‐436 cells were significantly lower in the AA treatment groups compared with controls (Figure 4D).

We next examined whether the antiproliferation effect of AA was via cell cycle arrest by performing flow cytometry analysis to test cell cycle distribution after AA treatment. After AA treatment, the proportion of cells in G2/M + S phase significantly increased (all *P* < .0001; Figure 4E). Furthermore, Annexin V‐FITC/PI assay showed that AA induced the apoptosis of MDA‐MB‐231 and MDA‐MB‐436 cells (Figure 4F).

### 3.4 AA inhibits breast cancer‐induced bone metastasis and osteolysis in vivo

**FIGURE 5:**
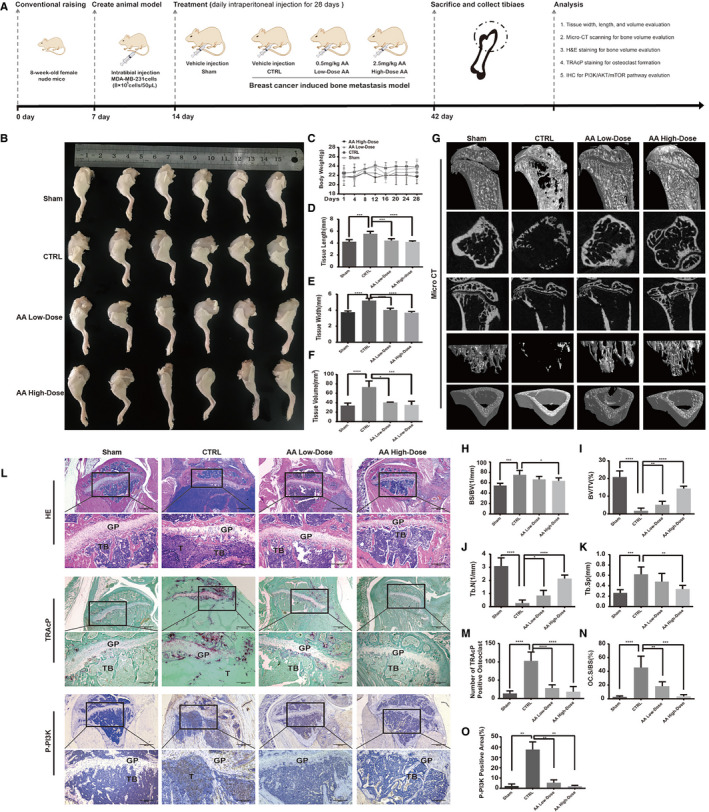
Asperolide A (AA) treatment inhibited breast cancer development and prevented breast cancer‐induced bone metastasis and osteolysis in vivo. Establishment of breast cancer‐induced bone metastasis nude mouse model and evaluation of AA effects (A). MDA‐MB‐231 cells were injected directly into the tibial plateau. AA inhibited tumor growth in the tibia (B). Body weight of mice (C). Tissue length of tibial tumor section (D). Tissue width of tibial tumor section (E). Tissue volume of tibial tumor section (F). Representative micro‐CT images indicated that AA prevented osteolysis (G). Quantitative analyses of bone structure parameters, including BS/BV (%), BV/TV (%), Tb.N, Tb.Sp (n = 6 per group) (H‐K). Representative tartrate‐resistant acid phosphatase (TRAcP) staining images showed that AA inhibited osteoclast formation (L). Quantitative analyses of TRAcP‐positive cell number (OC.N/BS) and area (OC.S/BS) (M, N). Representative immunohistochemistry images showed that AA inhibited the expression of P‐PI3K (L). Quantitative analyses of P‐PI3K positive area (%) (O). Data are shown as mean ± SD; n = 6 per group; *P < .05, ***P < .001, and ****P < .005, compared with the sham or CTRL groups

Based on our results, we next evaluated the effect of AA treatment on breast cancer‐induced osteolysis in vivo by establishing an experimental bone metastasis model. MDA‐MB‐231 cells were injected directly into tibiae plateau. Mice were divided into sham group, control group (CTRL group), AA high‐dose group, and AA low‐dose group. High‐dose mice were injected with AA (2 mg/kg) every day after model establishment, while low‐dose group mice were injected with AA (0.5 mg/kg) and CTRL group mice were injected with vehicle. There was no significant weight loss in the four groups (Figure 5C). After 28 days, we measured tumor formation. The tumors grew rapidly in CTRL group mice. However, the tumors in both high‐ and low‐dose AA‐treated groups grew significantly slower in a dose‐dependent manner (Figure 5D‐F). These results indicated that AA effectively suppressed breast cancer growth.

Micro‐CT analysis further revealed that AA prevented breast cancer cell‐induced osteolysis in bone metastasis model. Quantitative analysis showed that bone geometric parameters, including BS/BV (1/mm), BV/TV (%), TB.Sp, and Tb.N, were significantly increased in the AA treatment groups (Figure 5G‐K). As shown in Figure 5G, there was no clear bone trabecula structure in CTRL group mice. However, higher numbers of bone trabecular were found in AA treatment groups. TRAcP staining revealed that the total TRAcP‐positive osteoclast number per bone surface and Oc.S/BS were both decreased in AA‐treated groups compared with the CTRL group (Figure 5L‐N). The osteoclast and breast cancer‐related upstream factor, P‐PI3K, was also examined by IHC. The results showed that the expression of P‐PI3K was decreased in AA‐treated groups compared with untreated control group (*P*_AA‐high‐dose_ = .0012, *P*_AA‐low‐dose_ = .0022; Figure 5L,O). Together these analyses showed that AA can partially attenuate breast cancer bone metastasis and osteolysis.

## 4 DISCUSSION

Diterpenoids are a group of compounds with many pharmaceutical and industrial applications. Our group has been working on the evaluation of bioactivity of marine‐derived agents for many years. Recently, we found three natural tetranorlabdane diterpenoids with the same parent nucleus and different substituent group from a marine alga‐derived endophytic fungus *A wentii* that showed anticancer activities.^13^ Among the three compounds, AA and WB exhibited lower cytotoxicity on BMMs. The enhanced cytotoxicity of AA may be due to epoxy in position C1 and C2.^22^, ^23^ Compared with WB, AA had better anti‐osteoclast activity. The improvement in anti‐osteoclast activity may be due to C2‐C3 double bonds and the adjacent hydroxyl. Overall, our results demonstrated that AA showed the highest anti‐osteoclast activity and lowest cytotoxicity among all three compounds examined.

Our in vitro cell‐based assays showed for the first time that the marine‐derived agent AA inhibited osteoclasts formation and bone resorption. The anti‐osteoclastic effect of AA was not associated with cytotoxicity. BMMs treated with AA formed smaller and less osteoclasts than untreated controls by repressing ROS activities and F‐actin ring formation, suggesting impairment of precursor cell fusion. AA treatment also inhibited proliferation, migration, apoptosis, and invasion and induced G2M/S phase arrest of MDA‐MB‐231 and MDA‐MB‐436 cells. Our in vivo assays showed that AA suppressed breast tumor growth and protected breast cancer‐induced osteolysis.

**FIGURE 6:**
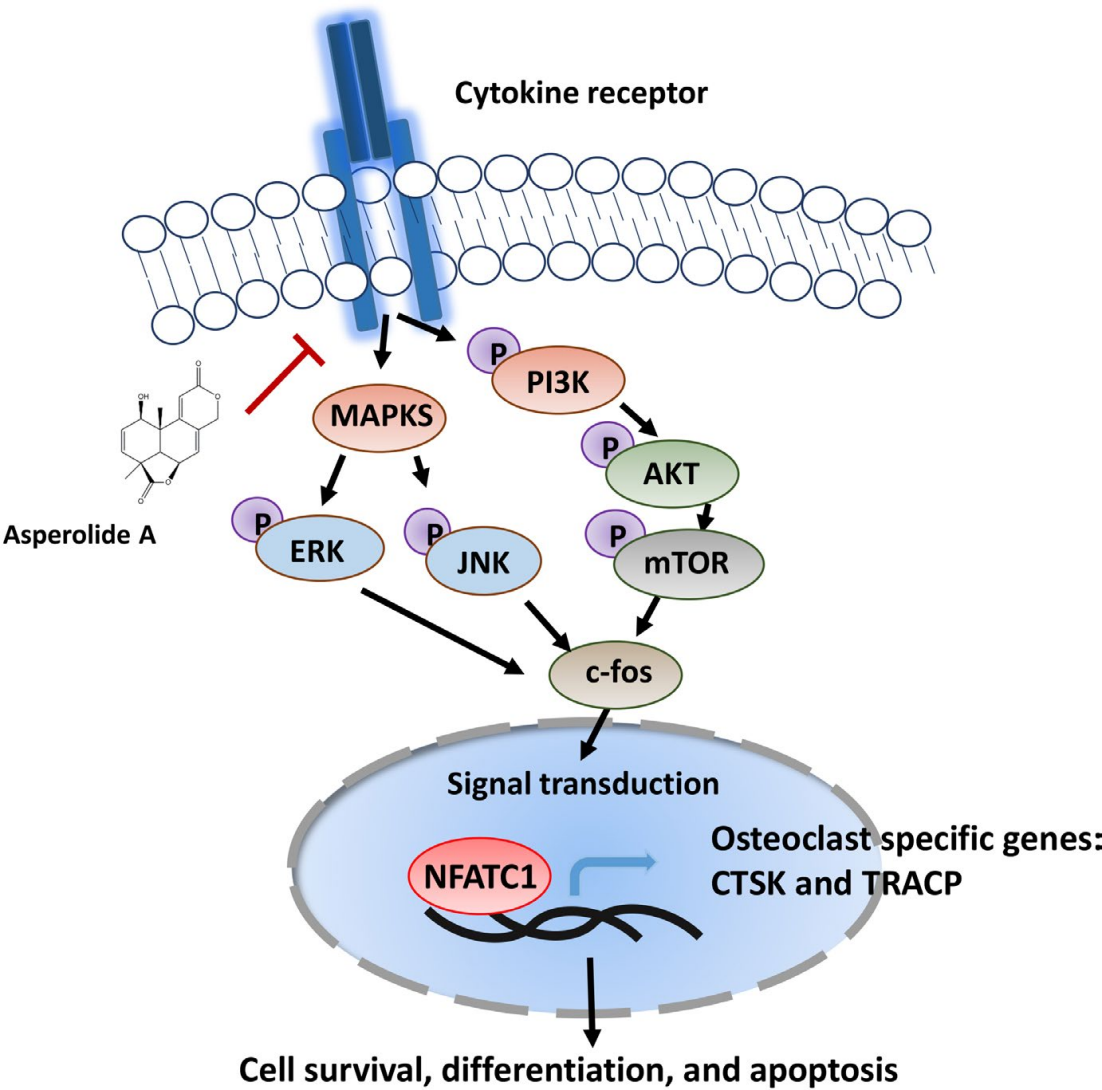
A potential working model for the suppression of Asperolide A in bone metastatic breast cancer

Accumulating evidence indicates that the PI3K/AKT/mTOR pathway plays an important role in osteoclast differentiation.^24^, ^25^, ^26^ In our study, western blot results indicated that AA inhibited the phosphorylation of PI3K, AKT, and mTOR. NFATc1 has been reported as the dominating transcriptional regulator of osteoclast differentiation, which is activated by c‐fos and plays an important role in cell migration, adhesion, and osteoclast‐mediated bone resorption.^27^, ^28^ In osteoclasts, the sequential activation of PI3K, AKT, and mTOR involves c‐Fos and NFATc1 proteins. Our results showed that the expression level of c‐Fos and NFATc1 following RANKL stimulation was hindered by AA. What's more, osteoclast‐specific genes, including c‐fos, Tracp, and Ctsk, which are directly regulated by NFATc1, were inhibited by AA (Figure 3A). Further investigation into the mechanisms revealed that AA significantly repressed the phosphorylation of JNK and ERK in response to RANKL. However, we did not observe any noticeable effects of AA on the activation phosphorylation of p65 and p38 (see Figure S1B). PI3K/AKT/mTOR is also the most frequently activated signaling pathway in breast cancer and promotes tumor growth and progression. Here, we also demonstrated that AA can inhibit PI3K/AKT/mTOR activation, leading to inhibited proliferation, inhibited migration, inhibited invasion, enhanced apoptosis, and arrest of G2M/S phase cell cycle in breast cancer cells as same as BEZ235 (inhibitor of PI3K/mTOR). Together these results suggest that AA hindered the phosphorylation of PI3K, AKT, mTOR, JNK, ERK, but not P65 or P38, suggesting that AA targets a specific upstream activator of MAPK and PI3K/mTOR signaling pathway (Figure 6). To investigate the potential targets of AA, Swiss TargetPrediction and Swissdock servers were used to search protein targets of AA and simulate AA‐target protein interaction at atomic level. Results showed that AA formed hydrogen bonds with the c‐KIT residues Tyr568 and Tyr570(PDB ID:4HVS), which is the active site inducing the activation of PI3K^29^ (see Figure S1D). However, further studies will need to be conducted to verify the interaction of AA and c‐KIT.

Further, we established a breast cancer‐induced bone metastasis model to investigate whether AA has therapeutic effect in vivo. We found that AA has a significant protective effect on breast cancer‐induced bone loss in a mouse model as confirmed by micro‐CT and H&E staining. Osteoclast formation was reduced by AA treatment as confirmed by Tracp staining, which is consistent with the result in vitro. In addition, P‐PI3K expression was downregulated in vivo in the AA treatment group.

In conclusion, this study has demonstrated for the first time that the natural compound AA can effectively inhibit osteoclastogenesis and prevent breast cancer‐induced bone osteolysis through suppression of the PI3K/AKT/mTOR signaling cascade.

## CONFLICT OF INTEREST

There is no conflict of interest.

## AUTHORS CONTRIBUTIONS

Wenli Jiang and Binghua Jiao conceptualized and designed the study. All the authors performed in vitro and in vivo experiments, involved in data analysis and interpretation, and also performed statistical analysis. Wenli Jiang, Youlutuziayi Rixiati, and Hao Huang drafted the manuscript. Binghua Jiao and Caiguo Huang involved in critical revision of the manuscript.

## Supporting information

Fig S1

Supplementary Material

## Data Availability

The data that support the findings of this study are available from the corresponding author upon reasonable request.
