# Supplementary figures and images for: Asperolide A prevents bone metastatic breast cancer via the PI3K/AKT/mTOR/c‐Fos/NFATc1 signaling pathway
Source: Cancer Med. 2020 Sep 25;9(21):8173–85. doi: 10.1002/cam4.3432 (PMC7643645; doi:10.1002/cam4.3432)

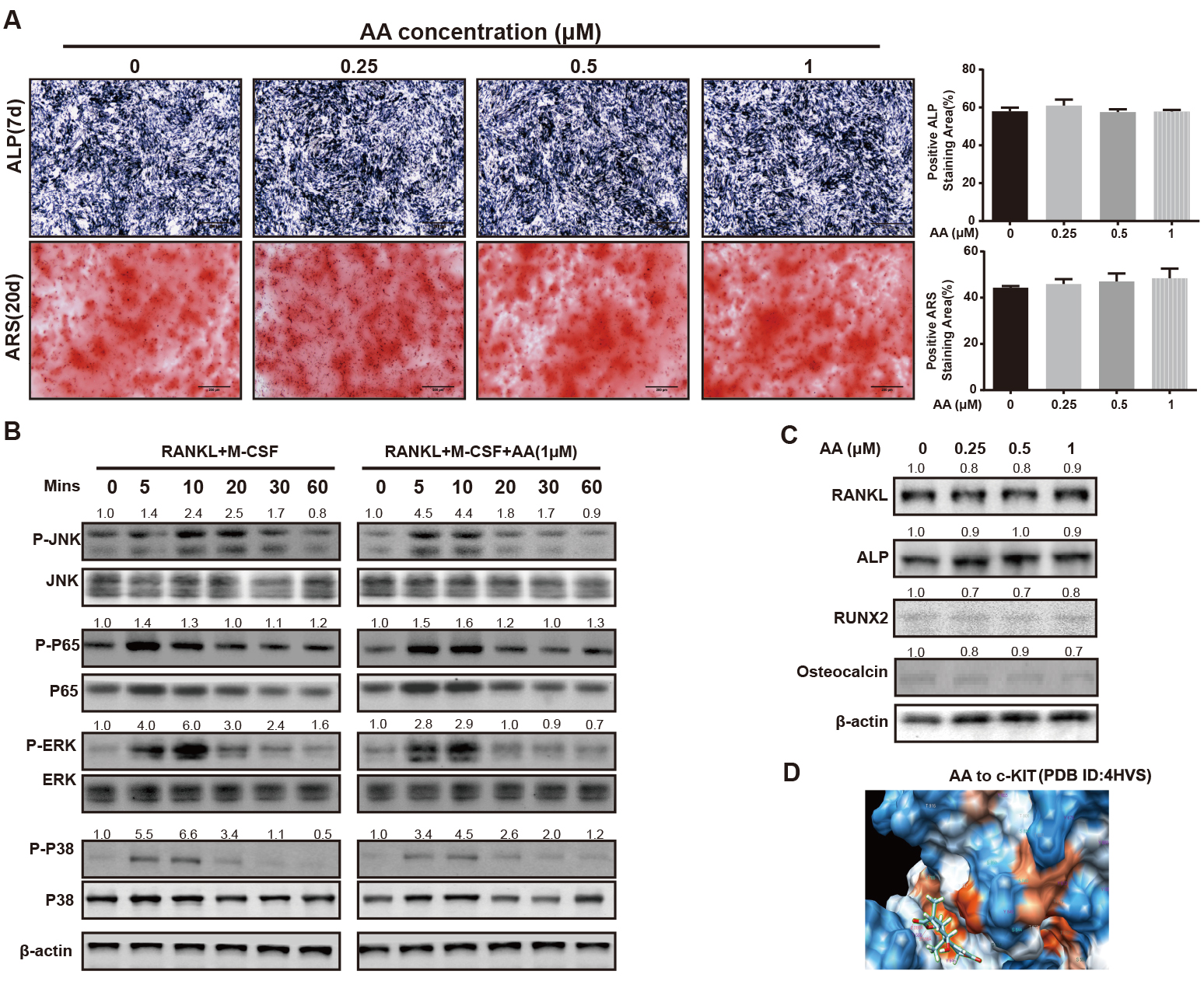

Supplement: Supplementary file 1 — Fig S1 [file CAM4-9-8173-s001.jpg]
