# Supplementary material for: Asperolide A prevents bone metastatic breast cancer via the PI3K/AKT/mTOR/c‐Fos/NFATc1 signaling pathway
Source: Cancer Med. 2020 Sep 25;9(21):8173–85. doi: 10.1002/cam4.3432 (PMC7643645; doi:10.1002/cam4.3432)
Supplement: Supplementary file 2 — Supplementary Material [file CAM4-9-8173-s002.docx]

**Figure S1.** AA does not affect osteogenic differentiation. ALP staining and ARS staining were performed after 7 days (ALP staining) and 21 days (ARS staining) with different concentrations of AA (A). M-CSF-dependent BMMs were plated in 6-well plates in starvation culture medium for 2h, pretreated without or with 1μM AA for 60 minutes, and then treated without or with 50 ng/ml RANKL for 5, 10, 20, 30, and 60 minutes. Western blot was performed to evaluate phosphorylated JNK (P-JNK), total JNK, phosphorylated P65 (P-P65), total P65, phosphorylated AKT (P-ERK), total ERK, and β-actin (C). Protein expression of ALP, Runx2, and osteocalcin in BMSCs without or with AA (0.25, 0.5, and 1μM) was assayed by western blot (C). AA interacted with c-KIT residues Tyr568 and Tyr570 (D).
